# Are EDs the Only Option? Hospital‐Based Alternatives to the Emergency Department for Mental Health Crises: A Scoping Review

**DOI:** 10.1111/inm.70060

**Published:** 2025-05-19

**Authors:** Carly Hudson, Candice Bowman, Marcus Randall

**Affiliations:** ^1^ Bond University Faculty of Health Sciences and Medicine Robina Queensland Australia; ^2^ University of New England Faculty of Medicine and Health Armidale New South Wales Australia; ^3^ Bond University Bond Business School Robina Queensland Australia; ^4^ Gold Coast Hospital and Health Service, Mental Health and Specialist Services Southport Queensland Australia

**Keywords:** crisis care, emergency department, health service evaluation, mental health services

## Abstract

Mental health continues to have a significant negative impact on global health. Hospital emergency departments (EDs) serve as a first entry point for individuals in crisis, with the number of presentations to EDs for mental health continuing to increase. However, EDs remain problematic environments for patients receiving emergency psychiatric care, due to the lack of suitable space, resources and specialised staff training. The World Health Organization has acknowledged the need to restructure mental health services to prioritise accessibility and person‐centred care. To address this need, a number of alternative crisis care services have been established, which provide short‐term emergency psychiatric care. This scoping review aims to provide an overview of the types of crisis services available within or adjacent to a hospital service. A systematic search of CINAHL, Medline, SocIndex and PsycINFO was conducted, returning 1213 results. Following title and abstract, and full text screening, 17 sources were included in the final review. Alternative crisis care services situated within or near existing hospital sites were broadly grouped into four categories: psychiatric emergency services, crisis stabilisation or observation units, specialised services for specific populations and non‐clinical crisis services. Of the included articles, 13 reported some form of service evaluation, examining a range of patient‐, staff‐ and service‐factors. Alternative crisis care services to the ED play a crucial role in providing accessible, localised support for individuals experiencing mental health crisis, potentially reducing the reliance on hospital‐based services. However, to date, there is a lack of consistency in service descriptions, and comprehensiveness of service evaluations. Standardised and more thorough reporting of crisis care services is required to better understand what services are available, and the impact they are having on mental health crisis care.

## Graphical abstract

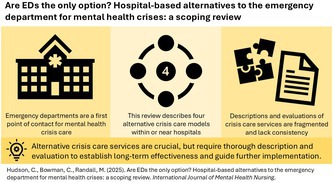

## 1 Introduction

The World Health Organization (WHO) estimated that globally in 2019, 970 million people lived with a mental health condition, and 703 000 people died by suicide, with these numbers expected to have increased since the COVID‐19 pandemic (World Health Organization 2022). This continued increase results in an urgent need for emergency psychiatric treatment services to support the needs of patients who are experiencing a mental health crisis. Increasingly, hospital emergency departments (EDs) are being used as a first point of contact for these patients to receive urgent and timely care (Australian Institute of Health and Welfare 2023; Brazel et al. 2023; Hudson et al. 2022).

Although EDs are a first entry point for patients requiring emergency care, EDs are problematic environments for patients receiving emergency psychiatric care. By design, they are generally set up to treat physical emergencies, resulting in a lack of suitable space, resources and specialised staff training to provide psychiatric care (Saurman et al. 2015). Patients have reported dissatisfaction with receiving mental health care in the ED and report excessive waiting times in an overstimulating and overcrowded environment (Clarke et al. 2014; Judkins et al. 2019; Zeller 2019). Patients presenting for mental health are twice as likely to leave without receiving treatment, resulting in patients who are at risk of harm to themselves or others returning to the community (State of Queensland (Queensland Health) 2020). Patients also perceived negative attitudes and stigma from ED staff, leading to patients feeling lonely, intimidated, ignored, uncomfortable, that seeking treatment at the hospital is a ‘waste of time’ (Shattell et al. 2014) and that the staff did not consider their presentation serious or urgent (Clarke et al. 2014). Individuals in crisis often just need a safe space to rest, reflect and make sense of their problem (Clarke et al. 2014; Shattell et al. 2014). The current ED environment does not provide the treatment environment needed to meet the needs of these individuals (Judkins et al. 2019).

Over the last 45 years, the provision of mental health care has shifted away from psychiatric hospitalisation to community care, resulting in a steady increase in available inpatient bed spaces, in favour of care within the community, with the aim of alleviating the pressure from acute psychiatric wards (Johnson 2013; Murphy et al. 2012; Saxon et al. 2018; Tyrer 2011). However, historically, community care was only helpful to maintaining individuals' mental health during stable periods, and not during acute or crisis situations (Murphy et al. 2012). This gap led to a revolving‐door situation, where individuals were discharged from hospital when stable, only to present to hospital again when they were in a crisis situation (Murphy et al. 2012). Individuals in crisis report having ‘nowhere else to go’, and in the midst of their distress, are often faced with the dilemma to stay home and hope that the symptoms will resolve, or to present to ED (Clarke et al. 2014; Heyland et al. 2013).

Consequently, the WHO has acknowledged the need to restructure mental health services to prioritise accessibility and person‐centred care, whilst maintaining integration of these services into existing health care services, such as hospitals and community health centres (World Health Organization 2022). To address this need, a number of alternative crisis care services have been established, which provide short‐term emergency psychiatric care, either within a community health centre or in a facility attached to an existing hospital. In these services, care is typically provided by a multidisciplinary team, with the aim of reducing unnecessary ED visits and hospitalisations, providing a positive patient experience, and resolving the mental health crisis in the short term (Balfour 2020; Zeller 2019, 2010).

Although individual service evaluations have been conducted, the authors' search of Open Science Framework (OSF) and PROSPERO has revealed that to date, there has not been an overall review of such alternative services for psychiatric crisis care within or near a hospital setting. A scoping review of hospital‐based alternatives to the ED is necessary to address this critical gap in the literature. Despite the increasing implementation of these services, there is a lack of comprehensive synthesis of the available services. Whilst individual service descriptions and evaluations exist, a scoping review is necessary to provide understanding of how these alternative services operate and fit within the broader healthcare system. Therefore, this scoping review aims to fill this gap by describing the types of crisis services available within or adjacent to a hospital service and providing an overview of patient‐, staff‐ and service‐related evaluations which have been conducted to date.

## 2 Method

This scoping review was conducted in accordance with the recommendations by Johanna Briggs Institute (JBI) (Peters et al. 2022) and the PRISMA extension for scoping reviews (PRISMA‐ScR) (PRISMA 2025).

### 2.1 Selection Criteria

The selection criteria were developed using the population (patients presenting for a psychiatric crisis), concept (alternative crisis care services to the ED) and context (in‐ or near‐hospital services) framework (Table 1). For the purpose of this review, alternative crisis care services are defined as services other than the ED, based within or near an existing hospital site, which aim to provide urgent care for individuals experiencing a mental health crisis.

**TABLE 1:** Inclusion and exclusion criteria.

Inclusion	Exclusion
Articles examined an alternative crisis care service Alternative crisis care services were situated within or near an existing hospital site Alternative crisis care services aimed to resolve the patients' mental health crisis in the short term Sources provided a description or evaluation of an existing alternative service (i.e., not general guidelines or recommendations)	Services: police street triage services, day hospitals, online or digital health services, residential psychiatric care services, home‐stay psychiatric care models, inpatient services, services without a focus on mental health crisis Articles that did not describe or evaluate alternative services Articles that only highlighted a need for alternative services, without description of a specific service Literature review articles

### 2.2 Search Strategy

A search strategy was developed with the assistance of a librarian who was experienced with health services research. CINAHL, Medline, SocIndex and PsycINFO were searched on 3 February 2025, using both MeSH terms and free‐text keywords. Both grey literature and empirical articles were included to ensure a broad coverage of the field. Grey literature was used as many services and models of service are described in documents such as government reports, rather than peer‐reviewed literature. Organisational websites were therefore also searched for grey literature that described crisis response models. The full search strategy is available in Appendix A.

### 2.3 Screening Process

The results from the database search were imported into Covidence, a systematic review management tool, where duplicate sources were removed (Covidence 2023). Articles were screened by two raters first by title and abstract, and then by full text by four raters. A total of 17 sources have been identified for inclusion in this review.

### 2.4 Data Extraction

A data extraction template was developed by two members of the research team (C.H. and C.B.) to chart data relating to study year and location, service location (in‐ or near‐hospital), service model, operating hours, staffing, referral types and any evaluation that had been reported. Data were extracted by two of the authors (M.R. and C.H.), and then synthesised for inclusion in this review.

## 3 Results

### 3.1 Selection of Sources

**FIGURE 1:**
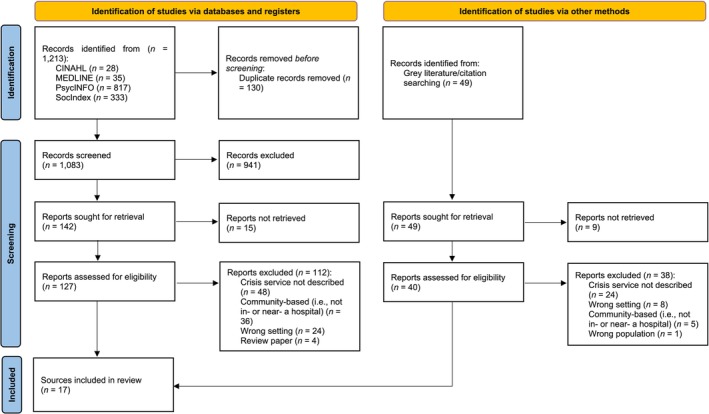
PRISMA flow diagram.

The database search returned a total of 1213 sources. Following de‐duplication, the titles and abstracts of 1083 sources were screened. A total of 941 sources were excluded as they did not meet eligibility criteria. The full texts of 127 sources were screened, resulting in the exclusion of 112 sources which did not meet the prespecified inclusion criteria. The final review included 17 sources relating to in‐ or near‐hospital crisis care services (Balfour 2020; Breslow et al. 1993; Currier and Allen 2003; Dion et al. 2010; Fitton and Reagan 2018; Francis et al. 2000; Frank et al. 2005; Gabet et al. 2020; Griswold et al. 2010; Hugo et al. 2002; JA Projects 2012; State of Victoria 2021; Mukherjee and Saxon 2019; Ruggeri et al. 2006; Thinn et al. 2015; State of Queensland (Queensland Health) 2020; Wolff 2008) (Figure 1).

### 3.2 Characteristics of Hospital‐Based Crisis Services

**FIGURE 2:**
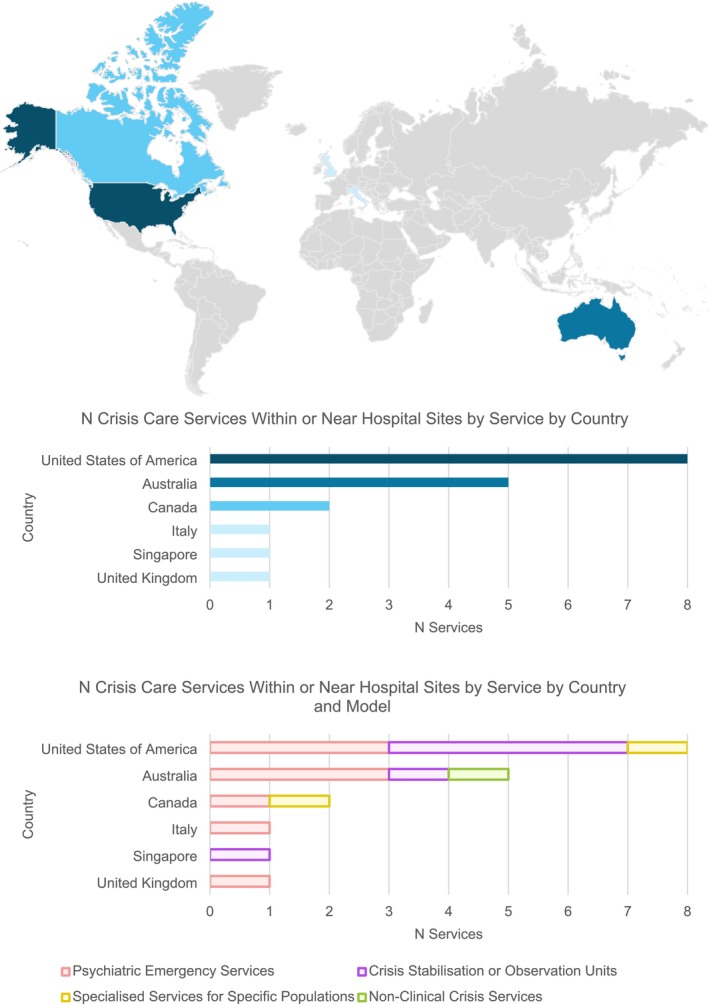
Number of crisis care services within or near hospital sites by service model and country.

The included sources were published between 1993 and 2020 (refer to Appendix B for the study matrix). Table 2 describes the key characteristics of these services. Eight of the included sources described psychiatric emergency services (Breslow et al. 1993; Currier and Allen 2003; Fitton and Reagan 2018; Frank et al. 2005; Gabet et al. 2020; Hugo et al. 2002; JA Projects 2012; Ruggeri et al. 2006), six sources described crisis stabilisation or observation units (Balfour 2020; Griswold et al. 2010; Mukherjee and Saxon 2019; Thinn et al. 2015; State of Queensland (Queensland Health) 2020; Wolff 2008), two described specialised services for specific populations (Dion et al. 2010; Francis et al. 2000) and one described a non‐clinical crisis service (State of Victoria 2021). The crisis services in the included studies were situated within the United States of America (*n* = 8) (Balfour 2020; Breslow et al. 1993; Currier and Allen 2003; Fitton and Reagan 2018; Francis et al. 2000; Griswold et al. 2010; Mukherjee and Saxon 2019; Wolff 2008), with five in Australia (Frank et al. 2005; Hugo et al. 2002; JA Projects 2012; State of Victoria 2021; State of Queensland (Queensland Health) 2020), two in Canada (Dion et al. 2010; Gabet et al. 2020), and one each in Italy (Ruggeri et al. 2006), Singapore (Thinn et al. 2015) and the United Kingdom (Ruggeri et al. 2006) (Figure 2).

**TABLE 2:** An overview of crisis response models and frameworks.

Model	Name of service	Country	Continuum of Care				Location		Operating hours		Staff		Referral	
			Prevention	Early intervention	Crisis response	Crisis resolution	Within existing hospital site	Near existing hospital site	24/7 basis	Restricted hours	Clinician‐led	Peer‐led	Self‐referral	Referral required
Psychiatric Emergency Services	Behavioural Health Emergency Services Unit	USA (Fitton and Reagan 2018)			✓	✓	✓		✓		✓		✓	
	Western Regional Assessment and Crisis Intervention Service	Australia (Hugo et al. 2002)			✓		✓			✓	✓		✓	
	Psychiatric Emergency Department, Services, Room, Centre or Care Centres	USA (Currier and Allen 2003, Breslow et al. 1993), Australia (Frank et al. 2005, JA Projects 2012), Canada (Gabet et al. 2020), Italy (Ruggeri et al. 2006)		✓	✓	✓	✓		✓		✓	✓	✓	✓
	24‐h Emergency Clinic	United Kingdom (Ruggeri et al. 2006)			✓	✓	✓		✓		✓			✓
Crisis Stabilisation or Observation Units	Crisis Response Center	USA (Balfour 2020)		✓	✓	✓		✓	✓		✓	✓	✓	✓
	Crisis Stabilisation Unit/Facility	USA (Mukherjee and Saxon 2019, Wolff 2008), Australia (State of Queensland (Queensland Health) 2020)			✓	✓	✓	✓	✓		✓	✓	✓	✓
	23‐h Observation Unit	Singapore (Thinn et al. 2015)			✓		✓		✓		✓			✓
	Comprehensive Psychiatric Emergency Program	USA (Griswold et al. 2010)			✓		✓		✓		✓			✓
Specialised Services for Specific Populations	Crisis Intervention Program	Canada (Dion et al. 2010)			✓	✓	✓			✓	✓		Not specified	
	Psychiatric Observation Program	USA (Francis et al. 2000)			✓	✓	✓		✓		✓		Not specified	
Non‐Clinical Crisis Services	Safe Haven Café	Australia (State of Victoria 2021)	✓	✓			✓	✓		✓		✓	✓	

Abbreviations: UK, United Kingdom; USA, United States of America.

#### 3.2.1 Continuum of Crisis Care

**FIGURE 3:**
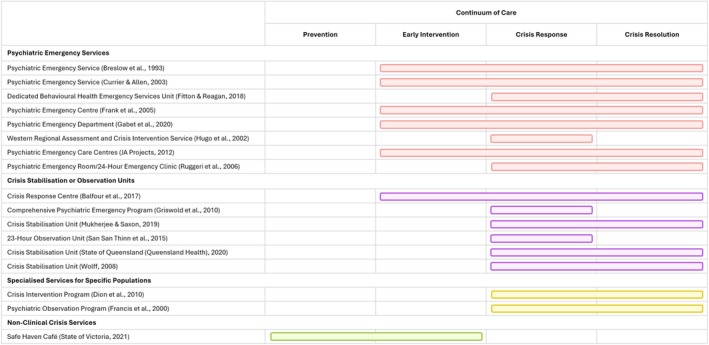
Continuum of crisis care for services within or near hospital sites.

The Continuum of Crisis Care describes which stage of crisis intervention each service is targeting (i.e., (i) prevention, (ii) early intervention, (iii) crisis response and (iv) crisis resolution) (State of Queensland (Queensland Health) 2020) (Figure 3). The majority of services target either crisis response (*n* = 10, 90.0%) (Balfour 2020; Breslow et al. 1993; Currier and Allen 2003; Dion et al. 2010; Fitton and Reagan 2018; Francis et al. 2000; Frank et al. 2005; Gabet et al. 2020; Griswold et al. 2010; Hugo et al. 2002; JA Projects 2012; Mukherjee and Saxon 2019; Ruggeri et al. 2006; Thinn et al. 2015; State of Queensland (Queensland Health) 2020; Wolff 2008) or crisis resolution (*n* = 7, 63.6%) (Balfour 2020; Breslow et al. 1993; Currier and Allen 2003; Dion et al. 2010; Fitton and Reagan 2018; Francis et al. 2000; Frank et al. 2005; Gabet et al. 2020; JA Projects 2012; Mukherjee and Saxon 2019; Ruggeri et al. 2006; State of Queensland (Queensland Health) 2020), with three services (27.2%) targeting early intervention (Balfour 2020; Breslow et al. 1993; Currier and Allen 2003; Frank et al. 2005; Gabet et al. 2020; JA Projects 2012; State of Victoria 2021; Ruggeri et al. 2006) and one (0.9%) targeting prevention (State of Victoria 2021).

#### 3.2.2 Service Factors

Of the 11 different services described (Table 2), most services were located within an existing hospital site (*n* = 9, 81.8%) (Breslow et al. 1993; Currier and Allen 2003; Dion et al. 2010; Fitton and Reagan 2018; Francis et al. 2000; Frank et al. 2005; Gabet et al. 2020; Griswold et al. 2010; Hugo et al. 2002; JA Projects 2012; Ruggeri et al. 2006; Thinn et al. 2015; State of Queensland (Queensland Health) 2020; State of Victoria 2021; Wolff 2008), with two services (18.2%) located nearby (Balfour 2020; Mukherjee and Saxon 2019). Eight (72.7%) of the services operated on a 24/7 basis (Balfour 2020; Breslow et al. 1993; Currier and Allen 2003; Fitton and Reagan 2018; Francis et al. 2000; Frank et al. 2005; Gabet et al. 2020; Griswold et al. 2010; JA Projects 2012; Mukherjee and Saxon 2019; Ruggeri et al. 2006; Thinn et al. 2015; State of Queensland (Queensland Health) 2020; Wolff 2008), whilst three (27.2%) operated with restricted hours (Dion et al. 2010; Hugo et al. 2002; State of Victoria 2021). Three services (27.2%) required referral (Griswold et al. 2010; Ruggeri et al. 2006; Thinn et al. 2015), three (27.2%) enabled self‐referral (Fitton and Reagan 2018; Hugo et al. 2002; State of Victoria 2021) and three (27.2%) allowed either referral or self‐referral (Balfour 2020; Breslow et al. 1993; Currier and Allen 2003; Frank et al. 2005; Gabet et al. 2020; JA Projects 2012; Mukherjee and Saxon 2019; Ruggeri et al. 2006; State of Queensland (Queensland Health) 2020; Wolff 2008). Two (11.8%) did not specify referral requirements (Dion et al. 2010; Francis et al. 2000).

**FIGURE 4:**
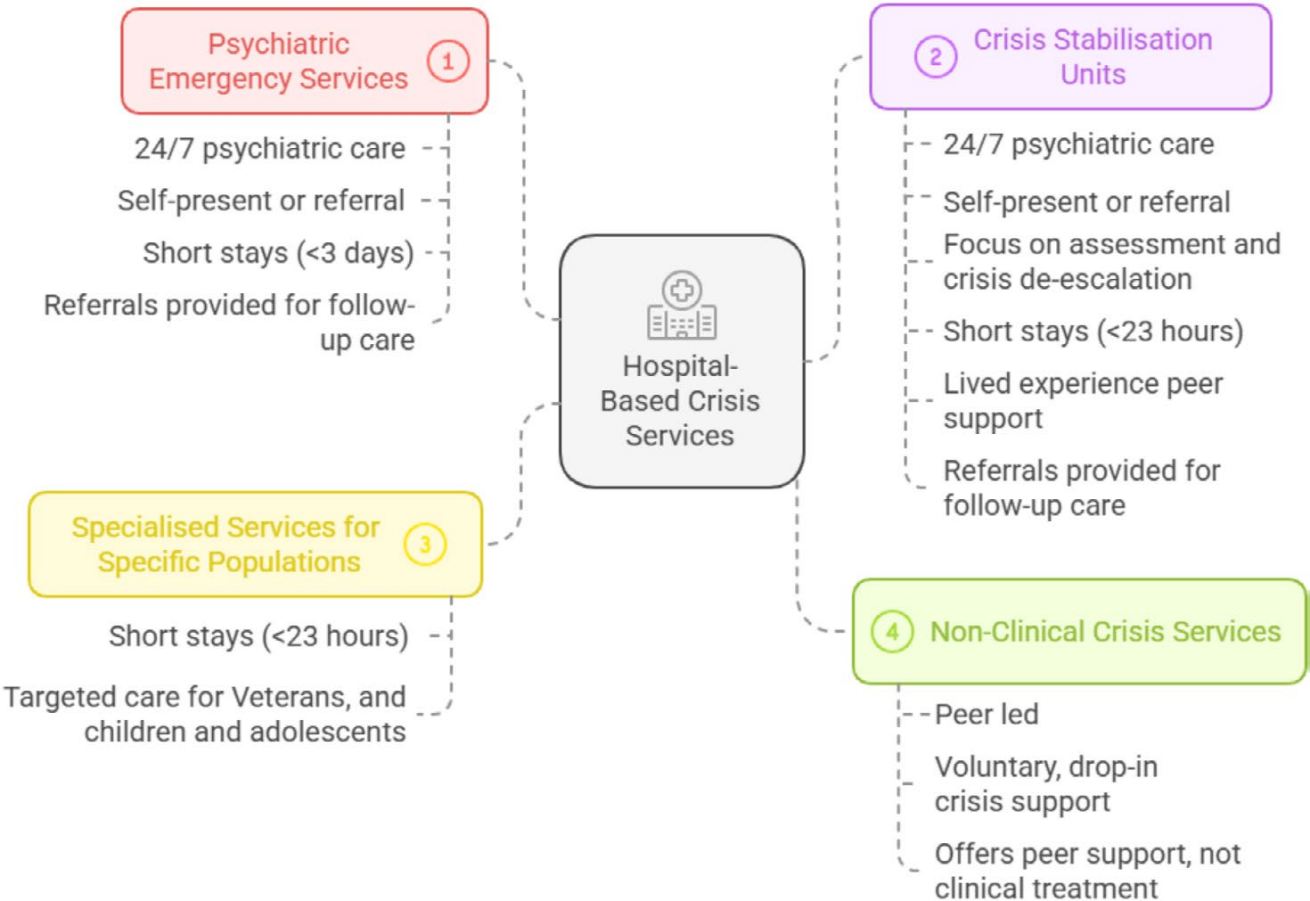
Characterisation of hospital‐based crisis services.

The services outlined in Table 2 show the diverse approaches to crisis care across the continuum, highlighting key characteristics of the models described. To enable analysis of these services, these have been broadly categorised into four distinct types: psychiatric emergency services (PES), crisis stabilisation or observation units (CSUs), specialised services for specific populations and non‐clinical crisis services (Figure 4).

### 3.3 Methods of Service Evaluation

The majority of included studies (*n* = 13, 76.5%) included some form of evaluation of the described crisis service (Table 3). The remaining four studies (*n* = 23.5%) did not provide any service evaluation. The evaluations that were conducted were diverse in terms of domains evaluated and methods of evaluation. Most frequently, evaluations examined patient factors (e.g., patient experience or satisfaction, patient characteristics and number of further hospitalisations), staff factors (e.g., number and type of staff, skillset, mental health‐related training) and service factors (e.g., clinical workflow and practices, treatment environment and cost‐effectiveness) were also examined for some services.

**TABLE 3:** Areas and methods of evaluating crisis services.

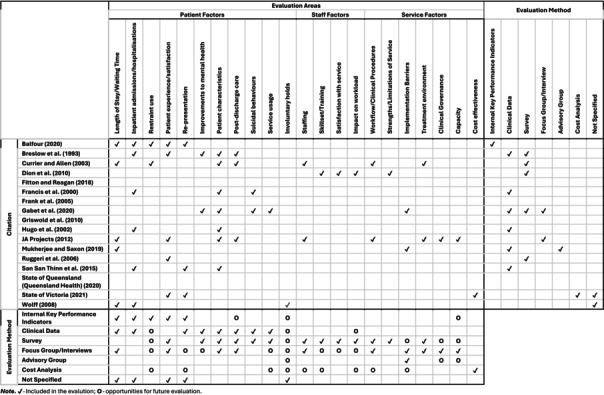

The methods of evaluation already conducted highlight several opportunities where future evaluations could be conducted. For instance, involuntary patient holds, restraint use, staff skillset and training, staff satisfaction with the service, impact of the service on staff workload, strengths and limitations of the service, clinical governance, capacity and cost effectiveness were the least evaluated domains and could be further explored in future evaluations. Clinical data and surveys were the most frequently used methods; however, internal key performance indicators, focus groups or interviews, advisory groups and cost‐analysis could all be used to triangulate evaluation data. Table 2 highlights particular opportunities where each method may be used to further evaluate specific domains.

### 3.4 Psychiatric Emergency Services

Psychiatric emergency services (PES) refer to observational units which offer psychiatric assessment and intensive treatment to both voluntary and involuntary patients who require urgent mental health care (Currier and Allen 2003). PES services are located within existing hospital sites, generally operate on a 24/7 basis and are primarily clinician‐led (e.g., psychiatrists, mental health nurses, social workers, counsellors and medical staff) with some services also involving peer‐support workers (Fitton and Reagan 2018; Gabet et al. 2020; Ruggeri et al. 2006; Hugo et al. 2002; JA Projects 2012). Patients can either self‐present or be referred by other healthcare staff such as clinicians in the general emergency department, police and ambulance services, or general practitioners (Frank et al. 2005; Ruggeri et al. 2006). PES can act as a major entry point into the mental health system, and can also act as a main treatment site for individuals with chronic mental illness (Currier and Allen 2003). PES prioritises short observation periods, usually lasting overnight or up to 3 days, to allow crises to resolve organically and reduce the need for inpatient admission (Breslow et al. 1993). Individuals in crisis receive psychiatric evaluation, psychopharmacology treatment (if clinically indicated), family intervention, counselling or psychotherapy, social work or financial support, and referral to ongoing services (Currier and Allen 2003). Individuals are observed for a short period of time in PES (usually overnight but stays can be extended to 2–3 days); this short observation period allows time for the crisis to resolve organically (as most mental health crises do) and thus avoids further inpatient admission (Breslow et al. 1993). Upon discharge, PES can link individuals to mobile crisis response teams or outpatient services to provide follow‐up care (Currier and Allen 2003). PES also can transfer individuals to inpatient admission for further care and specialist treatment, if needed (Breslow et al. 1993).

To date, the majority of evaluations that have been conducted on PES are focused on patient factors, including assessing the patient population, number of inpatient admissions or hospitalisations, patient experience and satisfaction, improvements to mental health, post‐discharge care, suicidal behaviours and service usage (Breslow et al. 1993; Currier and Allen 2003; Gabet et al. 2020; JA Projects 2012; Ruggeri et al. 2006). Patients reported using the PES services as they felt that other mental health services had not adequately responded to their needs or there was a lack of continuity of care (Gabet et al. 2020). Psychiatric symptoms reduced from the presentation to discharge, with 66.6% of surveyed patients reporting they felt better following their admission to the service (Breslow et al. 1993). Twenty‐two per cent of patients were reported to be placed on an involuntary hold following a voluntary admission, due to being at risk of harm to self or others or due to significant disability (Currier and Allen 2003). Mechanical restraint was reported to be used for 8.5% of patients (Currier and Allen 2003).

Overall, patients felt favourably towards the PES services and reported that the staff were compassionate, and that they were able to receive rapid treatment in a comfortable, friendly environment (Gabet et al. 2020; JA Projects 2012). However, patients still reported feeling negative stigma when waiting in the main hospital ED and were dissatisfied with the waiting time and location (JA Projects 2012). Carers who were surveyed also identified areas for improvements; carers stated that staff needed to listen to information that they provided and provide more information about what to do and who to contact in the case of a future crisis (JA Projects 2012).

When compared to patients' previous admissions to inpatient psychiatric wards, the PES service was rated as better or much better by 69.2% of patients. Usage of further mental health services decreased following a visit to the PES; Gabet et al. (2020) reported only 57% of patients used mental health services post‐discharge, in comparison with 72% prior to the PES service being established, whilst Breslow et al. (1993) reported only two of the 51 patients surveyed being re‐hospitalised in the 2 weeks following admission, and Currier and Allen (2003) reported that a third of patients presenting to the PES were admitted as psychiatric inpatients. Patients presenting with a personality disorder, or who were referred by community services were more likely to be discharged, whilst patients diagnosed with schizophrenia, who were assessed within the hospital (instead of the mobile‐based service), or with larger scores on the Psychiatric Symptom Assessment Scale, were more likely to be transferred for hospitalisation (Breslow et al. 1993; Hugo et al. 2002).

Staff within the PES services identified several challenges around providing patients with mental health care (Currier and Allen 2003; JA Projects 2012). Particularly in cases where an ED bed was not necessary, it was reported that there was limited space available to provide a thorough mental health assessment. JA Projects' (2012) evaluation identified several challenges between staff, such as the process for mental health assessment and medical clearance. In the described service, patients were required to be medically cleared prior to being admitted to the PECC; however, there were disagreements as to who should be responsible for this. Staff reported that although there were protocols in place for reporting and responding to domestic and family violence, child abuse and elder abuse, there were insufficient options for referring patients who presented with substance use (Currier and Allen 2003). Issues were also reported around the suitability of the treating environment, with a need to prioritise staff and patient safety. Additionally, the workflow between ED and the PECC was challenging, particularly when the patient was required to be transferred back to the PECC. It was therefore suggested that direct admission to the PECC, without the need to first attend ED, should be considered to streamline this process.

Interestingly, data collected during New South Wales Official Visitor Program visits in JA Projects' (2012) evaluation identified that in some cases, the PECCs were not being used for the intended purpose. Instead, they the PECCS were being used to manage overcrowding in the acute mental health wards, including admitting adolescents, which was not within their guidelines. The average length of stay (3.9 days) in these services was also found to exceed the maximum length of stay stated in the service guidelines (48 h). These findings suggest that the scope of the service needs to be routinely reviewed to ensure it meets the needs of both the patients and the wider health service.

### 3.5 Crisis Stabilisation or Observation Units

CSUs offer a safe environment for immediate crisis symptom relief and are an alternative to higher levels of care, such as inpatient admission (Francis et al. 2000; Thinn et al. 2015). The focus of these units is assessment, rapid stabilisation, de‐escalation of crisis and observation within a shorter time frame (maximum stay length of 23 h), with thorough discharge planning so that patients can continue to receive care once discharged back into the community (Mukherjee and Saxon 2019, Thinn et al. 2015, State of Queensland (Queensland Health) 2020, Wolff 2008, Balfour 2020). Patients are provided with a range of services including peer support, counselling, mental and physical evaluation, telepsychiatry, therapeutic intervention and ongoing assessment (Mukherjee and Saxon 2019, State of Queensland (Queensland Health) 2020, Wolff 2008). Patients can be either discharged following the crisis resolution, with discharge planning for follow‐up care; otherwise, an inpatient admission is facilitated (Francis et al. 2000; Gabet et al. 2020; Thinn et al. 2015).

The CSU services were found to be rated positively by patients, with Balfour (2020) reporting that over 85% of patients were likely to recommend the service, and Thinn et al. (2015) reporting that patients' mental health significantly improved following presentation. CSUs were found to be effective at reducing re‐hospitalisation or inpatient admission, with between 1.9% and 3% of patients returning within 72 h of discharge (Balfour 2020; Thinn et al. 2015), and 60%–70% of patients being diverted from inpatient services (Breslow et al. 1993). Patients who were older, self‐referred, had a past psychiatric history or had psychotic disorders were found to be more likely to be hospitalised (Thinn et al. 2015). Rates of involuntary holds and restraint use were also reduced (Balfour 2020; Wolff 2008).

Mukherjee and Saxon (2019) noted that although CSUs appear to be beneficial for patients' mental health, not all hospitals or health services have the resources, infrastructure or expertise to house an on‐site CSU. In this study, the CSU being examined acted as a central service for three nearby hospitals. This was found to be beneficial in providing a comfortable, welcoming environment, with lower rates of negative stigma than a CSU that was housed within a hospital site. However, the authors noted that the success of these services would be enhanced if patients were able to self‐present, rather than needing to first be medically cleared within the ED and then transferred.

### 3.6 Specialised Services for Specific Populations

Given that some populations require more specialised mental health care, some crisis services have been designed to provide targeted care to specific populations. Francis et al. (2000) described a 23‐h observation unit situated within a Veterans Affairs medical centre in the United States of America, where patients are provided with care by nursing staff, physicians and psychiatry staff to be observed and have their crisis stabilised. Similarly, Dion et al. (2010) described the Crisis Intervention Program (CIP), an ED‐based service dedicated to providing crisis care to children and adolescents.

Within the 23‐h Psychiatric Observation Program, 20 out of 92 (21.7%) of patients had an inpatient admission following their presentation to the observation unit (Francis et al. 2000). The unit was beneficial in reducing the average number of inpatient admission days, which decreased from 9.8 days to 2.7 days (Francis et al. 2000). Staff in the ED where the CIP was implemented were surveyed generally felt confident with dealing with, triaging and knowing when to refer patients to the crisis service, and were generally satisfied with the CIP service; however, felt there was a lack of availability of the Crisis Intervention Workers (CIWs) who provided the crisis interventions (Dion et al. 2010). Whilst staff felt that the CIP helped to reduce physician workload and appreciated the specialised training of the CIWs to manage and assess patients, they felt that the length of assessment and waiting time needed to be improved. Staff also identified that they would benefit from further training on mental health issues and how to assess patients with suicidal ideation (Dion et al. 2010).

### 3.7 Non‐Clinical Crisis Services

Non‐clinical crisis services provide emergency care at the earlier stages of the crisis continuum. For instance, the Safe Haven Café, located at St Vincent's Hospital in Victoria (Australia), provides non‐clinical care to patients in crisis who need assistance but not emergency care (State of Victoria 2021). Instead of focussing on psychiatric treatment, Safe Haven Cafes are led by lived experience peer workers and aim to provide patients with a safe space to share their stories and be heard. Individuals who present to the Safe Haven Café are supported by lived experience peer workers and mental health clinicians. Presentation to the Safe Haven Café is voluntary; however, individuals who present to the ED at St Vincent's Hospital may be referred by a peer worker (State of Victoria 2021).

Consumers who present to the Safe Haven Café display a high level of satisfaction, stating that it is a safe and welcoming environment for them to voice their concerns and receive support. Consumers report the open, collaborative environment as cathartic and appreciate the drop‐in model, which provides the opportunity to connect with other consumers experiencing similar problems (State of Victoria 2021). Following the opening of the Safe Haven Café, there was a significant reduction in mental health presentations to the ED, suggesting that this model is beneficial in diverting patients to alternative care options (State of Victoria 2021). Economically, the Safe Haven Café has resulted in a significant monetary benefit from avoided ED presentations, with each ED presentation in Victoria costing (at the time of reporting, for non‐admitted patients) approximately $600AUD (State of Victoria 2021).

## 4 Discussion

Emergency departments continue to be a frequently used, however challenging environment for the provision of emergency mental health care (Clarke et al. 2014; Judkins et al. 2019; Zeller 2019). In line with the WHO's recommendations to restructure mental health services, alternative options for crisis care are increasingly being established (World Health Organization 2022). The aim of this research was to describe the hospital‐based alternatives to the ED for mental health crisis care and to provide an overview of the evaluations that have been conducted on these services to date.

Whilst there was a range of different services examined in this review, to date, there is a lack of standardised definitions of what services entail, as evidenced by the inconsistencies in service descriptions found in this review. Although four models of care have been identified within this review, there is significant overlap in terms of service locations, staffing, processes and outcomes for patients, staff and service. The definitions of services in the literature remain ambiguous and heterogeneous, making it difficult to ascertain whether different services are indeed the same concept operating under a different service name. This ambiguity limits the ability to establish best practices and hinders the development of clear guidelines for service delivery.

To date, limited evaluation has been conducted on currently established crisis services. The studies which conducted some form of evaluation found that overall, crisis care services improved patients' mental health, with patients generally being satisfied with the services and finding them favourable to the ED or inpatient services (Balfour 2020; Breslow et al. 1993; Gabet et al. 2020; JA Projects 2012; Ruggeri et al. 2006; State of Victoria 2021). Accessing the alternative crisis services resulted in overall shorter waiting times, reduced length of stay and less negative stigma than those who presented to ED (Balfour 2020; Currier and Allen 2003; JA Projects 2012; Mukherjee and Saxon 2019; Wolff 2008). Despite the benefits of these services, several challenges and areas for improvement were identified. For instance, staff recognised the need for further training in mental health issues and assessment of suicidality (Currier and Allen 2003; JA Projects 2012). Issues around the workflow were also identified; for example, the process of providing patients with medical clearance, transferring patients between the ED and crisis care unit and whether or not patients were able to self‐present without first attending ED (Currier and Allen 2003). Resolving these issues and streamlining the crisis services is critical in ensuring the long‐term success of these alternative crisis care pathways.

The evaluations conducted to date have been fragmented and lack the comprehensiveness that is required to make meaningful comparisons and draw conclusions as to the effectiveness of crisis care services, particularly in comparison with traditional EDs. The variability in evaluation methods, data sources and focus (i.e., patients, staff or service) introduces further challenges when attempting to synthesise evaluations for crisis care models. These challenges limit the scalability of crisis care services and broader implementation efforts. To address these challenges, standardised evaluation reporting frameworks are required to ensure consistency in reporting across crisis care, and indeed other healthcare models. Piña et al. (2015) proposed a framework for describing healthcare organisations and systems, which encompasses service capacity (physical and human assets), organisational structure, finances, patients, care processes and infrastructure, and culture. Applying such a framework to crisis care models could facilitate meaningful comparisons between services, highlight areas for improvement, and inform policy and funding decisions.

### 4.1 Relevance to Clinical Practice

The emergence of alternative services to the ED for mental health crises is a positive move towards providing patients with comprehensive, patient‐centred care. These models aim to address the limitations of EDs by providing environments which are specifically designed for individuals in crisis, with suitable environmental design, staffing, resources and processes tailored to their unique needs. For clinicians, these services enable the rapid de‐escalation of the patient's mental health crisis and use a multidisciplinary approach to provide holistic care to address complex needs. Integrating these services into or near existing hospital sites can help to alleviate the burden on EDs, improve patient experience and outcome, and foster a compassionate and effective response to mental health emergencies. However, it is also necessary for standardised description and evaluation of these services, to enable meaningful comparisons and to understand the effectiveness as broader implementation is considered.

### 4.2 Limitations of the Current Study

The literature described in this review highlighted that crisis care services are currently a poorly described system of care, wherein similar services are referred to by vastly differing names, leading to confusion and lack of consistency. Descriptions of services are fragmented, and limited evaluation results in an inability to make meaningful comparisons between services. Although this review has been able to provide a summary on the types of crisis services currently described in the literature, our sources included both empirical and non‐empirical research, which varied in design and nature. It is likely that additional crisis services are available; however, they are not described in the literature or were not sufficiently described to meet the inclusion criteria of this review, and therefore were not included in this review. The available data around evaluation of crisis care services is also limited. Although further internal evaluations may have been conducted, many results are not publicly available and therefore could not be included in this review.

### 4.3 Recommendations for Future Research

The synthesis of the literature reviewed above offers several directions for further consideration. Whilst these alternative models provide promising options to divert individuals from EDs, they have not been adequately described nor comprehensively evaluated. In particular, evaluation of staff‐ and service‐related factors is lacking, which is important to understand to ensure the feasibility of continuing to provide these services. Without a clear and holistic description and evaluation of these services, it is unclear how these services can be best developed to continue to support patients in crisis. Therefore, future research in this area should consider the use of a standardised reporting framework for healthcare services to adequately describe the service and enable clear comparisons.

Whilst these innovative crisis services can help individuals stabilise their symptoms and provide access to ongoing support in the community, crisis care is often fragmented and complex. Crisis services vary in terms of admission and inclusion criteria, location, hours of operation and type of care offered and service provider. There is therefore a need for integration and overarching coordination of crisis service provision within a region, with clarity of navigation for the person in crisis to the right care at the right time and place.

In addition to hospital‐based alternatives to the ED, there are also a myriad of community‐based crisis care services. Understanding how these hospital‐based models integrate with or complement community‐based crisis care models is an important avenue for future research to explore continuity of care as patients move across the crisis continuum.

## 5 Conclusions

Alternative crisis care services to the ED play a crucial role in providing accessible, localised support for individuals experiencing mental health crisis, potentially reducing the reliance on hospital‐based services. However, the current landscape of crisis care remains fragmented, with inconsistencies in service definitions, evaluation methods and integration with existing healthcare systems. Without a standardised approach to describing and assessing these models, it is difficult to establish best practices or determine their long‐term effectiveness. Future research must prioritise the use of comprehensive evaluation frameworks to facilitate meaningful comparisons, inform policy and guide implementation. By enhancing coordination between hospital‐based and community crisis services, healthcare systems can ensure that individuals receive timely, appropriate care, ultimately improving patient outcomes and reducing the burden on emergency departments.

## Author Contributions

**Carly Hudson:** conceptualization, data curation, formal analysis, investigation, project administration and writing (original draft, review and editing); **Candice Bowman:** conceptualization, data curation, formal analysis, project administration, resources, writing (original draft, review and editing) and supervision; **Marcus Randall:** data curation and writing (review and editing) supervision.

## Conflicts of Interest

The authors declare no conflicts of interest.

## Data Availability

The data that support the findings of this study are available from the corresponding author upon reasonable request.

## Appendix A Search Strategy

**Table:**

**CINAHL**	
S1	((MH ‘Mental Health’) AND (TI (acute OR emergency OR crisis) OR AB (acute OR emergency OR crisis))) OR (MH ‘Community Mental Health Services+’)
S2	(MH ‘Crisis Intervention’) OR TI (‘crisis intervention models’ OR ‘Alternatives to Emergency Department’ OR ‘Mental Health crisis intervention unit’ OR ‘St Vincent's Hospital Safe Haven Café’ OR ‘Redland Hospital *Living Edge’ OR ‘*crisis cafés’ OR ‘psychosocial support’ OR ‘peer workers’ OR ‘alternative care pathways’) OR AB (‘crisis intervention models’ OR ‘Alternatives to Emergency Department’ OR ‘Mental Health crisis intervention unit’ OR ‘St Vincent's Hospital Safe Haven Café’ OR ‘Redland Hospital *Living Edge’ OR ‘*crisis cafés’ OR ‘psychosocial support’ OR ‘peer workers’ OR ‘alternative care pathways’)
S3	(MH ‘Emergency Service+’) OR TI (‘ED’ OR ‘emergency department’ OR ‘emergency services’) OR AB (‘ED’ OR ‘emergency department’ OR ‘emergency services’)
S4	S1 AND S2 AND S3
**Medline**	
S1	((MH ‘Mental Health’) OR (MH ‘Community Mental Health Services’)) AND (TI (acute OR emergency OR crisis) OR AB (acute OR emergency OR crisis))
S2	(MH ‘Crisis Intervention’) OR TI (‘crisis intervention models’ OR ‘Alternatives to Emergency Department’ OR ‘Mental Health crisis intervention unit’ OR ‘St Vincent's Hospital Safe Haven Café’ OR ‘Redland Hospital *Living Edge’ OR ‘*crisis cafés’ OR ‘psychosocial support’ OR ‘peer workers’ OR ‘alternative care pathways’) OR AB (‘crisis intervention models’ OR ‘Alternatives to Emergency Department’ OR ‘Mental Health crisis intervention unit’ OR ‘St Vincent's Hospital Safe Haven Café’ OR ‘Redland Hospital *Living Edge*’ *OR ‘*crisis cafés’ OR ‘psychosocial support’ OR ‘peer workers’ OR ‘alternative care pathways’)
S3	(MH ‘Emergency Service, Hospital+’) OR TI (‘ED’ OR ‘emergency department’ OR ‘emergency services’) OR AB (‘ED’ OR ‘emergency department’ OR ‘emergency services’)
S4	S1 AND S2 AND S3
**SocIndex**	
S1	DE ‘mental health’ OR SU ‘community mental health services’ AND (TI (acute OR emergency OR crisis) OR AB (acute OR emergency OR crisis))
S2	DE ‘mental health’ OR DE ‘Crisis intervention (Mental health services)’ OR TI (‘mental health’ AND (‘crisis intervention’ OR ‘crisis intervention models’)) OR AB (‘mental health’ AND (‘crisis intervention’ OR ‘crisis intervention models’)) OR TI (‘Alternatives to Emergency Department’ OR ‘Mental Health crisis intervention unit’ OR ‘St Vincent's Hospital Safe Haven Café’ OR ‘Redland Hospital Living Edge’ OR ‘crisis cafés’ OR ‘psychosocial support’ OR ‘peer workers’ OR ‘alternative care pathways’) OR AB (‘Alternatives to Emergency Department’ OR ‘Mental Health crisis intervention unit’ OR ‘St Vincent's Hospital Safe Haven Café’ OR ‘Redland Hospital Living Edge’ OR ‘crisis cafés’ OR ‘psychosocial support’ OR ‘peer workers’ OR ‘alternative care pathways’)
S3	TI (‘hospital emergency service’ OR ‘ED’ OR ‘emergency department’ OR ‘emergency services’) OR AB (‘hospital emergency service’ OR ‘ED’ OR ‘emergency department’ OR ‘emergency services’)
S4	S1 AND S2 AND S3
S5	DE ‘COVID‐19 pandemic’ OR TI ‘COVID‐19’ OR AB ‘COVID‐19’
S6	S4 NOT S5
S7	acute OR emergency OR crisis
S8	S6 AND S7
**PsycINFO**	
S1	((‘Mental Health’/ AND (TI (acute OR emergency OR crisis) OR AB (acute OR emergency OR crisis))) OR ‘Community Mental Health Services+’/)
S2	‘Crisis Intervention’/ OR (TI (‘crisis intervention models’ OR ‘Alternatives to Emergency Department’ OR ‘Mental Health crisis intervention unit’ OR ‘St Vincent's Hospital Safe Haven Café’ OR ‘Redland Hospital Living Edge’ OR ‘crisis cafés’ OR ‘psychosocial support’ OR ‘peer workers’ OR ‘alternative care pathways’) OR AB (‘crisis intervention models’ OR ‘Alternatives to Emergency Department’ OR ‘Mental Health crisis intervention unit’ OR ‘St Vincent's Hospital Safe Haven Café’ OR ‘Redland Hospital Living Edge’ OR ‘crisis cafés’ OR ‘psychosocial support’ OR ‘peer workers’ OR ‘alternative care pathways’)))
S3	(‘Emergency Service+’/ OR TI (ED OR ‘emergency department’ OR ‘emergency services’) OR AB (ED OR ‘emergency department’ OR ‘emergency services’))
S4	S1 AND S2 AND S3

## Appendix B Study Matrix

**Table:**

Citation	Study title	Country	Name of service
Balfour (2020)	Using Lean to Rapidly and Sustainably Transform a Behavioural Health Crisis Program: Impact on Throughput and Safety	United States of America	Crisis Response Centre (CRC)
Breslow et al. (1993)	Crisis hospitalization on a psychiatric emergency service	Canada	Psychiatric Emergency Service
Currier and Allen (2003)	Organization and function of academic psychiatric emergency services	United States of America	Psychiatric Emergency Service
Dion et al. (2010)	Evaluating crisis intervention services for youth within an emergency department: a view from within	Canada	Crisis Intervention Program
Fitton and Reagan (2018)	Behavioral health crisis services–models and issues	United States of America	Dedicated Behavioural Health Emergency Services Unit
Francis et al. (2000)	Utilization and outcome in an overnight psychiatric observation program at a Veterans Affairs Medical Center	United States of America	Psychiatric Observation Program
Frank et al. (2005)	Development of Australia's first psychiatric emergency centre	Australia	Psychiatric Emergency Centre
Gabet et al. (2020)	Implementation of three innovative interventions in a psychiatric emergency department aimed at improving service use: a mixed‐method study	Canada	Psychiatric Emergency Department
Griswold et al. (2010)	A randomized trial: are care navigators effective in connecting patients to primary care after psychiatric crisis?	United States of America	Comprehensive Psychiatric Emergency Program
Hugo et al. (2002)	A comparison in hospitalization rates between a community‐based mobile emergency service and a hospital‐based emergency service	Australia	Western Regional Assessment and Crisis Intervention Service
JA Projects (2012)	External Review of Psychiatric Emergency Care Centres in NSW	Australia	Psychiatric Emergency Care Centres
Mukherjee and Saxon (2019)	“Psychological Boarding” and community‐based behavioral health crisis stabilization	United States of America	Crisis Stabilisation Unit
Ruggeri et al. (2006)	Satisfaction with community and hospital‐based emergency services amongst severely mentally ill service users: a comparison study in South‐Verona and South‐London	Italy; United Kingdom	Psychiatric Emergency Room; 24‐H Emergency Clinic
Thinn et al. (2015)	The 23‐h observation unit admissions within the emergency service at a national tertiary psychiatric hospital: clarifying clinical profiles, outcomes, and predictors of subsequent hospitalization	Singapore	23‐H Observation Unit
State of Queensland (Queensland Health) (2020)	Gold Coast Crisis Reform: A Strategic Approach to Transforming Mental Health Crisis Care	Australia	Crisis Stabilisation Unit
State of Victoria (2021)	Royal Commission into Victoria's Mental Health System—final report	Australia	Safe Haven Café
Wolff (2008)	Development of a psychiatric crisis stabilization unit	United States of America	Crisis Stabilization Unit
